# Usefulness of three-dimensional image navigation system for evaluation of hepatic artery before living donor liver transplantation: a case report

**DOI:** 10.1186/s40792-017-0359-2

**Published:** 2017-07-28

**Authors:** Michinori Matsumoto, Shigeki Wakiyama, Hiroaki Shiba, Yuichi Ishida, Yoshiaki Kita, Katsuhiko Yanaga

**Affiliations:** Division of Hepato-Biliary-Pancreatic Surgery, Department of Surgery, The Jikei University School of Medicine, 3-25-8 Nishi-shinbashi, Minato-ku, Tokyo, 105-8461 Japan

**Keywords:** Three-dimensional computed tomography, Computed tomographic angiography, Three-dimensional image, Region-growing method

## Abstract

**Background:**

The evaluation of the hepatic vascular anatomy in living liver donors is increasingly being performed by three-dimensional (3D) computed tomography (CT) angiography. However, details of hepatic artery anatomy obtained by 3D CT angiography are not always superior to those obtained by angiography. Here, we report a case in which the 3D image navigation system helped to detect segment II, III, and IV arteries (A2, A3, and A4, respectively) that individually originated from the proper hepatic artery (PHA); this could not be detected by 3D CT angiography.

**Case presentation:**

A 46-year-old man with end-stage primary biliary cirrhosis was admitted to our hospital for evaluation as a candidate for living donor liver transplantation. The patient’s younger sister, aged 43 years, was the only living donor candidate. The predicted left liver graft volume with the middle hepatic vein was found to be 403 mL using the region-growing method with 3D CT software. This volume was sufficiently large for the recipient because the standard liver volume of the recipient was 1095 mL. 3D CT angiography was performed twice but could not reveal the anatomical structure of the left and middle hepatic arteries. However, simulation using the region-growing method demonstrated individual branching off of A2, A3, and A4 from the PHA; conventional angiography demonstrated the same results. Each branch was approximately 1 mm in diameter, which was too small for secure anastomosis. Therefore, we selected the right liver graft for simplicity. The postoperative course of the donor and recipient was uneventful, and they were discharged on postoperative days 10 and 46, respectively.

**Conclusions:**

In conclusion, reconstruction of the hepatic vasculature using the 3D software by region-growing method might be a useful adjunct for surgical planning in the evaluation of the hepatic arteries in living liver donors.

## Background

The preoperative evaluation of donors for living donor liver transplantation (LDLT) aims to select a suitable donor with optimal graft quality and to ensure donor safety. Owing to extensive research on and rapid technical development of computed tomography (CT) scanners and three-dimensional (3D) workstations [1], the evaluation of hepatic vascular anatomy in living liver donors is increasingly being performed by 3D CT angiography. However, details of hepatic artery anatomy obtained by the 3D CT angiography are not always superior to those obtained by angiography [2]. On the other hand, 3D image navigation software systems enable not only the calculation of total liver volume and the volume of each vessel’s (both the portal and hepatic venous branches) territory but also the visualization of the anatomy of the donor’s hepatic vessels [3], which facilitates preoperative surgical planning. However, to our knowledge, the usefulness of these 3D image navigation software systems for evaluating the anatomy of the hepatic segmental arteries has not yet been reported.

Here, we describe a case in which the 3D image navigation system could detect the segment II, III, and IV arteries (A2, A3, and A4) that individually originated from the proper hepatic artery (PHA); this phenomenon could not be detected by 3D CT angiography.

## Case presentation

A 46-year-old Japanese man with end-stage primary biliary cirrhosis was admitted to our hospital for evaluation as a candidate for LDLT. On admission, the model for end-stage liver disease (MELD) score was 20, and the updated Mayo risk score was 11.3. The patient’s younger sister, aged 43 years, was the only living donor candidate. Her height and body weight were 153 cm and 57 kg, respectively.

Multidetector row CT (Siemens Somatom Definition Flash, Siemens Healthcare Japan, Tokyo, Japan) was employed for preoperative dynamic CT. As a contrast material, iopamidol (Iopamiron 370, Bayer, Tokyo, Japan) with an iodine concentration of 370 mg I/mL was intravenously administered (600 mg I/kg, maximum dose of 150 mL) over 30 s. Images in the arterial phase were obtained by the bolus tracking technique, and the scanning began 15 s after the trigger threshold, which was set at 80 HU above the aortic baseline CT number, was reached.

**Fig. 1:**
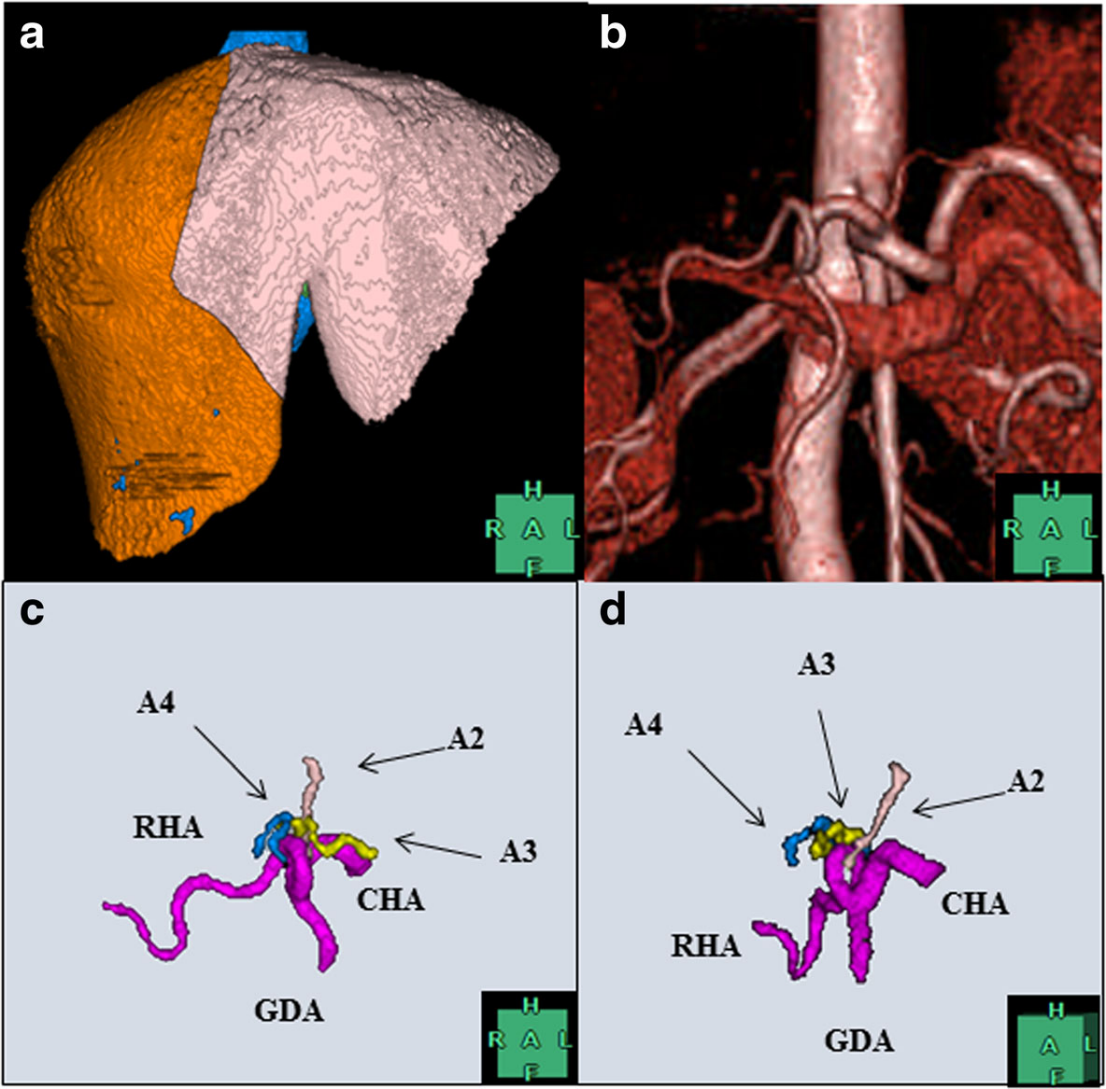
Donor hepatectomy simulation using the 3D CT software by region-growing method, based on dynamic helical CT of the liver. **a** The predicted volume of the total liver was 1056 mL, consisting of the right liver (653 mL) and the left liver with the middle hepatic vein (403 mL). **b** The 3D CT angiography showing no anatomical structure of the LHA and MHA. The simulation using the 3D CT software by region-growing method showing the segment II, III, and IV arteries (A2, A3, and A4 with *arrows*, respectively) individually originating from the proper hepatic artery. Images of the artery in **c** the front view and **d** the left anterior oblique view. *CHA* common hepatic artery, *GDA* gastroduodenal artery, *RHA* right hepatic artery

**Fig. 2:**
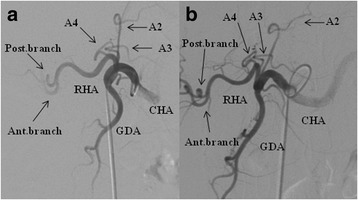
Angiography showing the segment II, III, and IV (A2, A3, and A4 with *arrows*, respectively) arteries individually originated from the proper hepatic artery like the simulation. In **a** the front view and **b** the left anterior oblique view. *CHA* common hepatic artery, *GDA* gastroduodenal artery, *RHA* right hepatic artery, *Post. branch* posterior branch of RHA, *Ant. branch* anterior branch of RHA

Figure 1a shows a simulation of donor hepatectomy, which was performed using the region-growing method with the 3D CT software (organ volume analysis (OVA), Liver Segmentation Software, Hitachi Medico, Tokyo, Japan) [3, 4]. The predicted left liver graft volume with the middle hepatic vein (MHV) was 403 mL, which was sufficiently large for the recipient because a ratio of the graft volume to the standard liver volume of the recipient (1095 mL) was ≥35%. 3D CT angiography was performed twice but could not reveal the anatomical structure of the left and middle hepatic arteries (LHA and MHA) in either case (Fig. 1b). The degree of contrast enhancement was quantified by measuring HU values by positioning the region of interest at the aorta, right hepatic artery (RHA), umbilical portion of the portal vein, and anatomical structures, such as the LHA or MHA, which were 363.9 ± 21.1, 304.1 ± 20.4, 123.8 ± 24.0, and 148.8 ± 26.8 HU, respectively. Because CT numbers of anatomical structures, such as the LHA or MHA, were much lower than those of the aorta or the RHA and were comparable to those of the surrounding umbilical portion of the portal vein, the LHA and MHA were deleted in the process of constructing the image of 3D CT angiography. An experienced radiologist and several surgeons suspected the LHA and MHA to have individually originated from the PHA. Subsequently, the hepatic arteries were also extracted using the region-growing method similar to the method used for extracting the liver parenchyma, portal vein, and hepatic vein: after setting a seeding point, the region was extended to neighboring voxels if the CT density of the voxel was within a specified range, which resulted in A2, A3, and A4 individually branching off from the PHA (Fig. 1c, d). To achieve the aforementioned diagnostic goal, conventional angiography was performed, which demonstrated that A2, A3, and A4 individually originated from the PHA, as demonstrated by 3D CT software (Fig. 2). Each branch was approximately 1 mm in diameter, which was considered as too small for secure anastomosis. If we had selected the left liver as graft with the MHV, we would have had to anastomose up to three branches. Therefore, the donor’s right liver was selected. The postoperative course of the donor and recipient was uneventful, and they were discharged on postoperative days 10 and 46, respectively.

### Discussion

The incidence of three pedicles from the LHA was 3.4% in the left liver graft [5]. Although successful segmental resection and anastomosis for the three pedicles from the LHA of living donors has been reported [5], we selected the right liver graft for simplicity.

3D image of A2, A3, and A4 could not be reconstructed for the following reasons: (1) firstly, low enhancement of A2, A3, and A4: one of the most important parameters to minimize partial volume effects in improving spatial resolution in CT angiography is a contrast density of >250 HU, which enables a continuous visualization of the arteries as small as 0.5 mm in diameter [6]. In the current case, the CT numbers of the aorta and RHA, which could be successfully reconstructed in the 3D image, were >250 HU, whereas those of the anatomical structures, such as the LHA or MHA, were considerably <250 HU. (2) Secondly, slow injection speed of the contrast material: in a comparison of an infusion rate of 2 mL/s with 4 mL/s, the visualization rates of the segmental hepatic arteries improved from 11–34% at 2 mL/s to 76–89% at 4 mL/s [2]. In the current case, the infusion rate was 3.1 mL/s, and A2, A3, and A4 were not regarded as extrahepatic but as segmental arterial branches. These segmental branches were deleted in the process of constructing the 3D CT angiography image, even when the infusion rate was 4 mL/s, because of the poor continuous arterial enhancement similar to that in a previous study [2]. Other less invasive modalities are warranted to comprehend the hepatic arterial anatomy.

The advantages of OVA using the region-growing method with 3D CT software are as follows: (1) 3D imaging of liver structures, (2) detailed volumetric analyses based on portal perfusion, and (3) quantitative estimates of the venous drainage area, enabling the investigation of unknown fields that cannot be examined using conventional two-dimensional modalities such as hand-tracing volumetry [3, 4]. According to the manufacturer’s information for OVA, the software is based on an algorithm that defines the perfusion area of each portal branch based on the direction and diameter of vessels [3, 4]. In the present case, the region-growing method was applied to the 3D image reconstruction of the hepatic arteries. Compared with the extraction of the arteries using semi-automatic tracing of the arteries, that using the region-growing method produced a more objective 3D image because extracted arterial images were reconstructed based on the CT densities of the voxels within the specified range of the hepatic arteries and not of the surrounding portal vein. The reconstruction of 3D image of these small less-contrasted branches using the region-growing method was visually successful.

## Conclusions

In conclusion, the reconstruction of hepatic vasculature using the region-growing method with 3D CT software might be a useful adjunct for surgical planning in the evaluation of the hepatic arteries in live liver donors.

## Abbreviations

3D: Three-dimensional
CT: Computed tomography
HU: Hounsfield unit
LDLT: Living donor liver transplantation
LHA: Left hepatic artery
MELD: Model for end-stage liver disease
MHA: Middle hepatic artery
MHV: Middle hepatic vein
PHA: Proper hepatic artery
RHA: Right hepatic artery
